# Effects of branched-chain amino acids on *Shiraia* perylenequinone production in mycelium cultures

**DOI:** 10.1186/s12934-023-02066-6

**Published:** 2023-03-24

**Authors:** Wen Hao Shen, Rui Peng Cong, Xin Ping Li, Qun Yan Huang, Li Ping Zheng, Jian Wen Wang

**Affiliations:** 1 College of Pharmaceutical Sciences, Soochow University, Suzhou, 215123 China; 2 Department of Horticultural Sciences, Soochow University, Suzhou, 215123 China

**Keywords:** *Shiraia bambusicola*, Branched-chain amino acids, Perylenequinones, Hypocrellin A, Eliciting

## Abstract

**Background:**

Perylenequinones from *Shiraia* fruiting bodies are excellent photosensitizers and widely used for anti-cancer photodynamic therapy (PDT). The lower yield of *Shiraia* perylenequinones becomes a significant bottleneck for their medical application. Branched-chain amino acids (BCAAs) not only serve as important precursors for protein synthesis, but also are involved in signaling pathway in cell growth and development. However, there are few reports concerning their regulation of fungal secondary metabolism. In present study, the eliciting effects of BCAAs including l-isoleucine (l-Ile), l-leucine (l-Leu) and l-valine (l-Val) on *Shiraia* perylenequinone production were investigated.

**Results:**

Based on the analysis of the transcriptome and amino acid contents of *Shiraia* in the production medium, we revealed the involvement of BCAAs in perylenequinone biosynthesis. The fungal conidiation was promoted by l-Val treatment at 1.5 g/L, but inhibited by l-Leu. The spore germination was promoted by both. The production of fungal perylenequinones including hypocrellins A (HA), HC and elsinochromes A-C (EA–EC) was stimulated significantly by l-Val at 1.5 g/L, but sharply suppressed by l-Leu. After l-Val treatment (1.5 g/L) in *Shiraia* mycelium cultures, HA, one of the main bioactive perylenequinones reached highest production 237.92 mg/L, about 2.12-fold than that of the control. Simultaneously, we found that the expression levels of key genes involved in the central carbon metabolism and in the late steps for perylenequinone biosynthesis were up-regulated significantly by l-Val, but most of them were down-regulated by l-Leu.

**Conclusions:**

Our transcriptome analysis demonstrated that BCAA metabolism was involved in *Shiraia* perylenequinone biosynthesis. Exogenous BCAAs exhibit contrasting effects on *Shiraia* growth and perylenequinones production. l-Val could promote perylenequinone biosynthesis via not only enhancing the central carbon metabolism for more precursors, but also eliciting perylenequinone biosynthetic gene expressions. This is the first report on the regulation of BCAAs on fungal perylenequinone production. These findings provided a basis for understanding physiological roles of BCAAs and a new avenue for increasing perylenequinone production in *Shiraia* mycelium cultures.

**Graphical Abstract:**

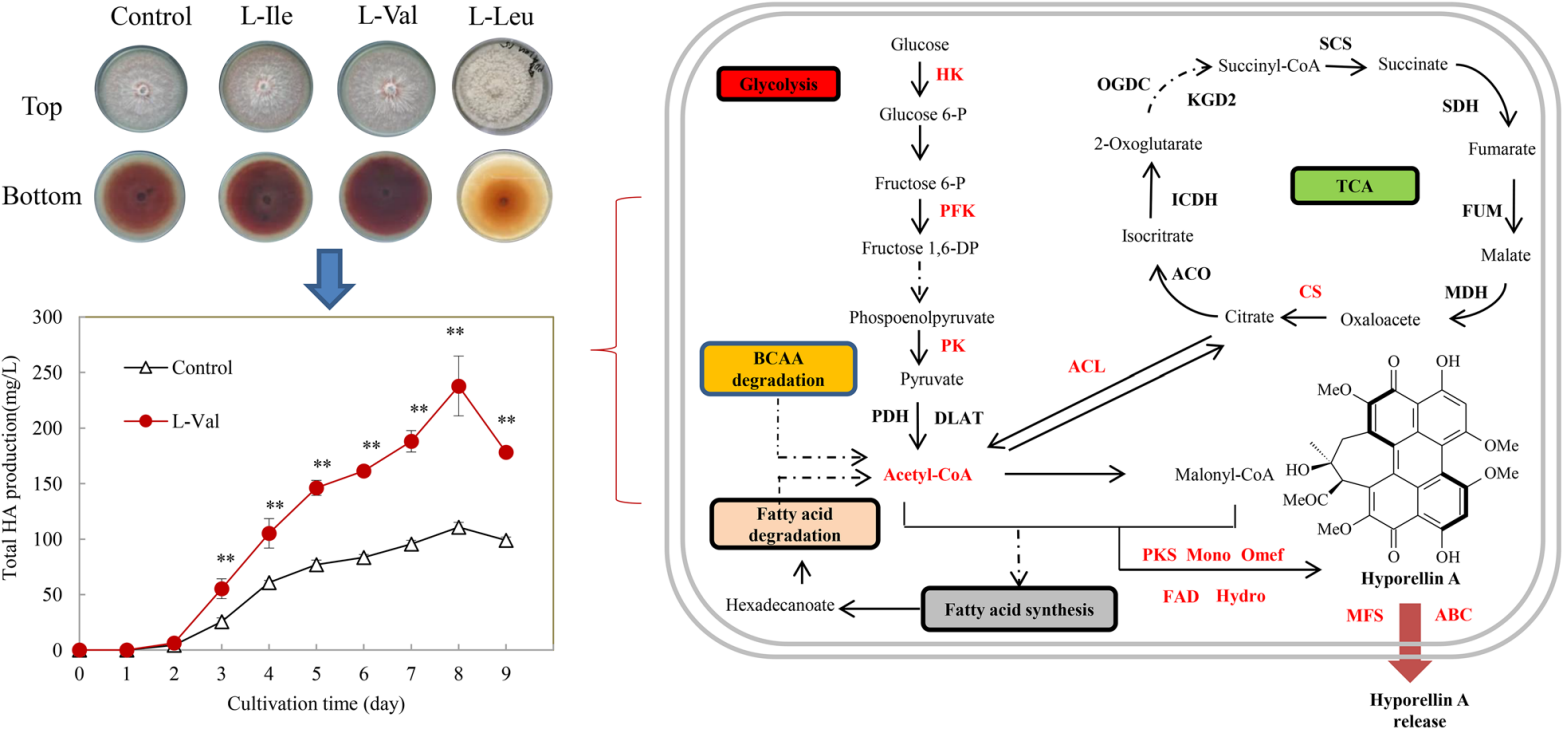

**Supplementary Information:**

The online version contains supplementary material available at 10.1186/s12934-023-02066-6.

## Background

The perylenequinone-rich *Shiraia* fruiting bodies have long been used as traditional Chinese medicine to treat vitiligo, stomachache, psoriasis and rheumatic arthritis [1]. The photosensitive perylenequinones are mainly isolated from the fruiting bodies of bambusicolous parasitic *Shiraia* fungi, including hypocrellin A–D (HA-HD) and elsinochrome A–C (EA-EC) [2]. Hypocrellins were main bioactive perylenequinones and developed as new photosensitizer in photodynamic therapy (PDT) on cancers, viruses and skin diseases [3]. Due to the difficulties of the chemical synthesis and artificial cultivation of *Shiraia* fruiting bodies [4], mycelium culture is becoming a biotechnological alternative for bioactive perylenequinone production [5]. However, the lower yield of perylenequinones in solid-state fermentation (HA 2.02 mg/g dry weight, DW) or in liquid fermentation (HA 10–40 mg/L and elsinochromes 9–74 mg/L) is becoming a bottleneck for their medicinal application [6–8].

The supply of precursors is critical for perylenequinone production as acetyl-CoA and malonyl-CoA were used to catalyze the formation of intermediate metabolite *nor*-toralactone [9]. A large amount of CoA products are derived from branched-chain amino acids (BCAAs) including isoleucine (Ile), leucine (Leu) and valine (Val) [10]. Exogenous BCAAs were often used to improve the production of macrocyclic polyketide antibiotics such as glycopeptide A40926, biotechspiramycin and pikromycin by *Streptomyces* [11–13]. It was also found that the overexpressing of a branched chain α-keto acid dehydrogenase (BCDH) for BCAA catabolism resulted in about 52-fold increase of actinorhodin production of *S. coelicolor* [14]. However, less is known regarding the regulatory roles of BCAAs on fungal metabolites.

The abiotic elicitation methods including adding surfactants, a lower intensity ultrasound, red light radiation and light/dark shifting were employed to improve perylenequinone production in *S. bambusicola* [15–19]. As there was no or lower concentrations of HA detected in submerged *Shiraia* cultures in the base medium, Triton X-100 was previously screened to induce hypocrellin production [15, 20]. The addition of 0.6% Triton X-100 to submerged cultures of *Shiraia* sp. SUPERH168 increased the production of total hypocrellins to 780.6 mg/L [20]. In our previous study [15], Triton X-100 at 2.5% (w/v) induced HA contents in both mycelia and medium. Therefore, Triton X‑100 is becoming the normal component in production medium for *Shiraia* perylenequinone production [15, 20, 21]. In this study, the involvement of BCAAs in the perylenequinone biosynthesis was revealed by the transcriptomic analysis of *Shiraia* cultures in the production medium supplemented with Triton X-100 at 2.5% (w/v) [15]. The effects of exogenous BCAAs (l-Val, l-Leu and l-Ile) on fungal growth and perylenequinone production of *Shiraia* cultures were observed. The contrasting regulation of l-Val and l-Leu on expression changes of biosynthetic genes for perylenequinones was investigated. In *Shiraia* mycelium cultures, the time and concentrations of l-Val addition was optimized to enhance HA production. We reported for the first time on the regulation of BCAAs on fungal perylenequinone metabolism. This study may help us understand the relationship between BCAAs and perylenequinone biosynthesis, and provide a new strategy to improve perylenequinone production in mycelium cultures.

## Result

### Transcriptomic changes related to BCAA metabolism and BCAA contents in *Shiraia* cultures for perylenequinone production

**Fig. 1:**
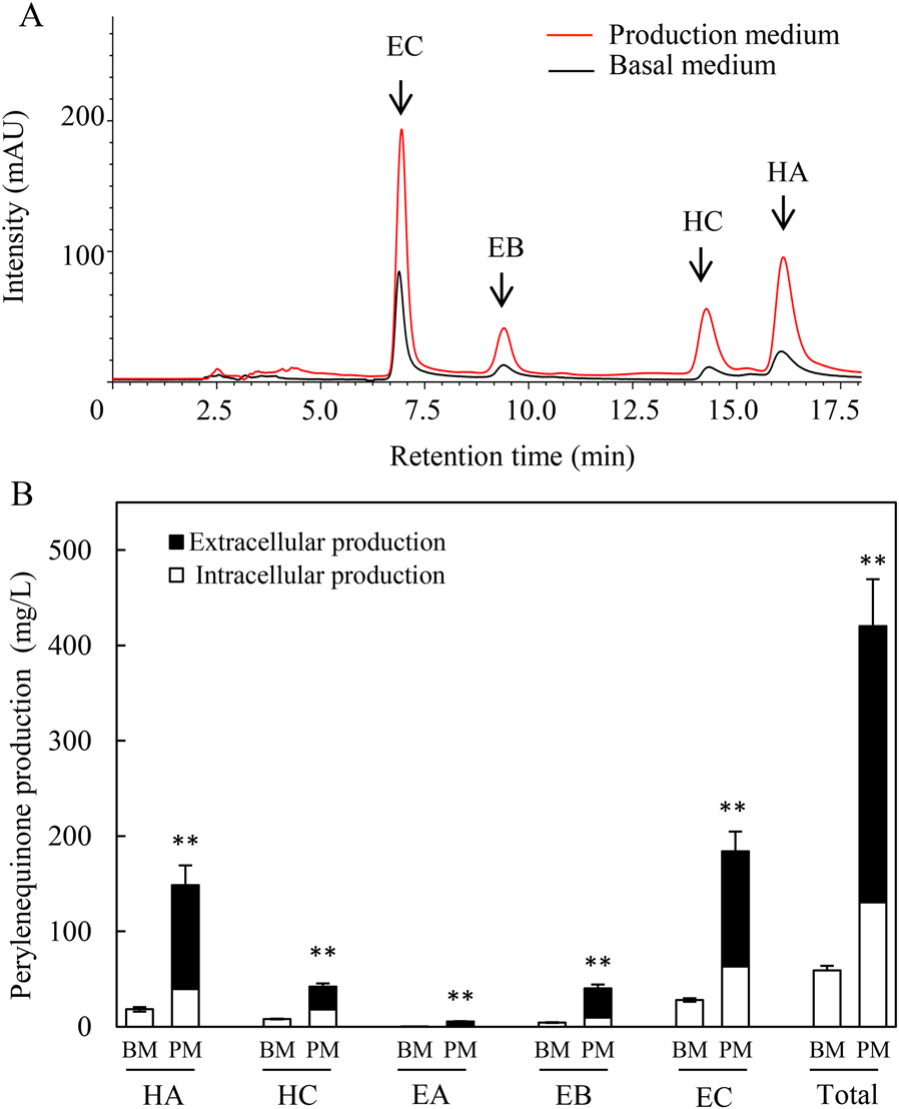
Perylenequinone production of *S. bambusicola* S8 in the basal medium (BM) without Triton X-100 and production medium (PM) with Triton X-100. **A** The HPLC chromatogram of perylenequinone production in *Shiraia* mycelium culture. **B** The individual perylenequinone production of *S. bambusicola* S8 in the basal medium (BM) and production medium (PM). S8 was cultivated at 150 rpm and 28 °C, and harvested on day 8. Values are mean ± SD from three independent experiments (^*^p < 0.05 and ^**^p < 0.01 versus control)

**Fig. 2:**
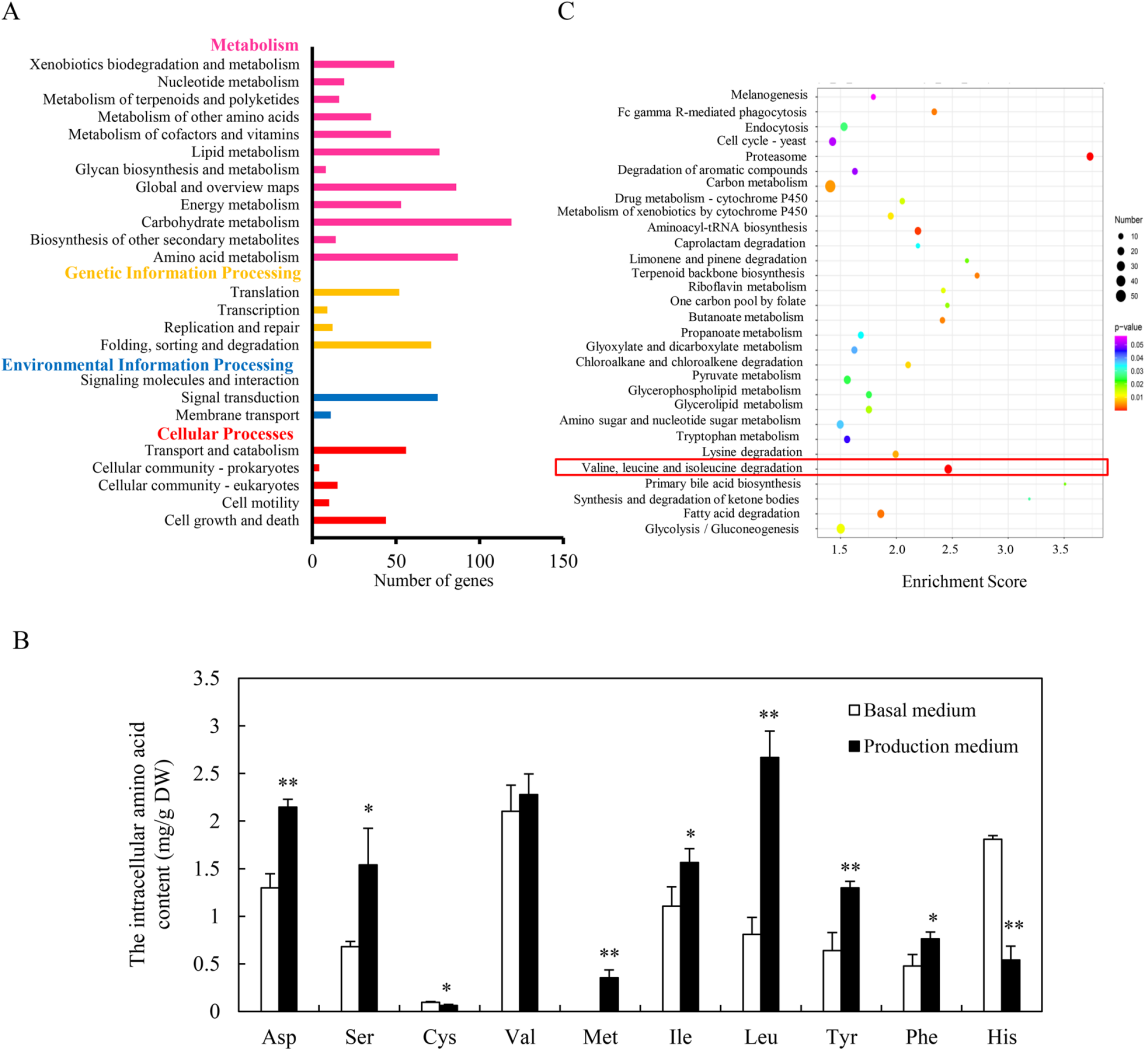
KEGG pathway analysis by RNA-Seq of fungus *S. bambusicola* S8 in the basal medium without Triton X-100 and production medium with Triton X-100. **A** DEGs mainly focused on Metabolism, Environmental Information Processing, Genetic Information Processing and Cellular Processes. **B** The intracellular amino acid contents of *S. bambusicola* S8 in the basal medium and production medium. **C** Top 30 of pathways in KEGG pathways. *S. bambusicola* S8 was harvested on day 8. The basal medium was used as control. Data shown is the mean ± SD (n = 3). Asterisks represent significant differences when compared to control group (^*^p < 0.05 and, ^**^p < 0.01 versus control)

**Fig. 3:**
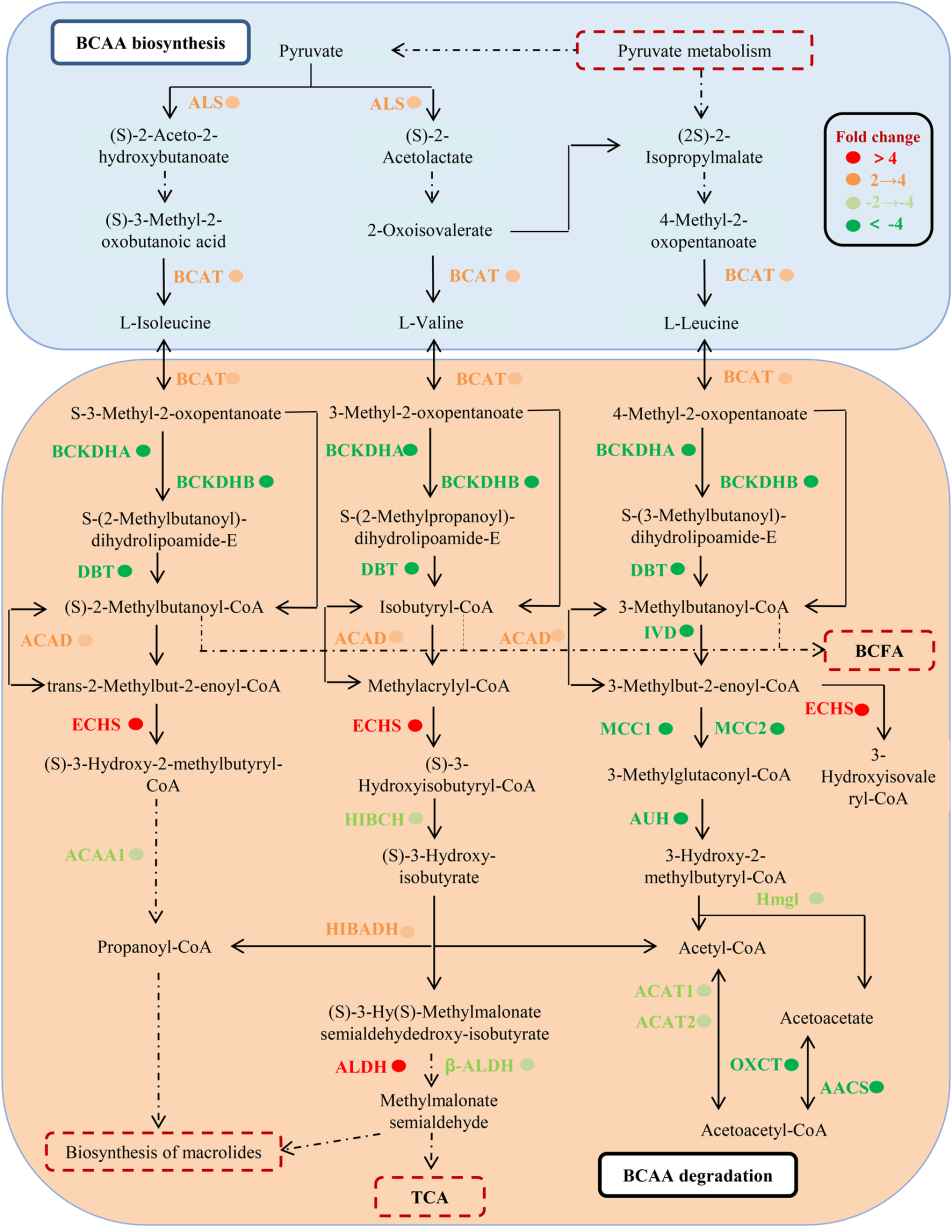
Network of metabolic pathways of BCAA metabolism of fungus *S. bambusicola* S8 cultured in the basal medium without Triton X-100 and production medium with Triton X-100. The transcripts with fold change (FC) ≥ 2 and *p* value ≤ 0.05 were selected. Genes shown in red and green were identified in KEGG database. Red represents the up-regulated genes, whereas green represents the down-regulated genes in the production medium compared with basal medium. Some of steps and compounds are omitted for simplification. The mycelial samples were collected on day 8. More information about enzyme and annotations are given in Additional file 1: Table S2

The comparison of *Shiraia* perylenequinone production in the basal medium without Triton X-100 and in the production medium with Triton X-100 was conducted (Fig. 1). The individual perylenequinone (HA, HC, EB and EC) was promoted significantly after 8-day cultures in the production medium containing Triton X-100 at 2.5% (w/v) (Fig. 1A). The released perylenequinones from the mycelia were induced only in the production medium (Fig. 1B). The total perylenequinone production in *Shiraia* culture in the production medium increased to 420.22 mg/L, about 7.13-fold of that in the basal medium without Triton X-100 (Additional file 1: Table S1). Based on the transcriptomic data (BioProjectPRJNA323638) from *S. bambusicola* S8 in our previous study [15], we analyzed corresponding KEGG (Kyoto Encyclopedia of Genes and Genomes) pathways of differential gene expression between *Shiraia* cultured in the basal medium and in production medium with Triton X-100 (Fig. 2). KEGG enrichment showed that the majority of DEGs were found to be involved in the category of “Metabolism”, such as “Carbohydrate metabolism” (119, 21.10%), “Global and overview maps” (86, 15.25%) and “Amino acid metabolism” (87, 15.43%) (Fig. 2A). In the production medium with Triton X-100, most of intracellular amino acid contents of *S. bambusicola* S8, such as l-Leu, l-Ile, l-aspartic acid (Asp) and l-tyrosine (Tyr) were significantly higher than that in the basal medium (Fig. 2B). Among them, l-Leu (2.67 mg/g) is the most abundant amino acid in *Shiraia* hyphae, followed by l-Val (2.28 mg/g), then l-Asp (2.15 mg/g), l-Ile (1.57 mg/g) and l-Ser (1.54 mg/g) in the production medium with Triton X-100. Furthermore, the most enriched terms for top 30 KEGG entries were found to be related to “Proteasome”, “Carbon metabolism” and “l-Val, l-Ile and l-Leu degradation” (Fig. 2C). These results suggested that the changes of amino acids, in particular BCAAs (l-Leu, l-Val, and l-Ile) could be important for *Shiraia* perylenequinone biosynthesis. We performed a further analysis on 26 DEGs encoding BCAA biosynthesis and degradation (Additional file 1: Table S2). In the biosynthetic pathways for l-Ile, l-Val and l-Leu (Fig. 3), the transcriptional levels of acetolactate synthase (ALS) and BCAA aminotransferase (BCAT) were increased by 1.7-fold and 2.7-fold, respectively (Additional file 1: Table S2). Simultaneously, the expression of some unigenes for BCAA degradation were also up-regulated, including acyl-CoA dehydrogenase (ACAD), enoyl-CoA hydratase (ECHS), aldehyde dehydrogenase (ALDH) and 3-hydroxyisobutyrate dehydrogenase (HIBADH). These results indicated an active BCAA metabolism for *Shiraia* perylenequinone production.

### The effects of exogenous BCAAs on fungal growth and perylenequinone production

**Fig. 4:**
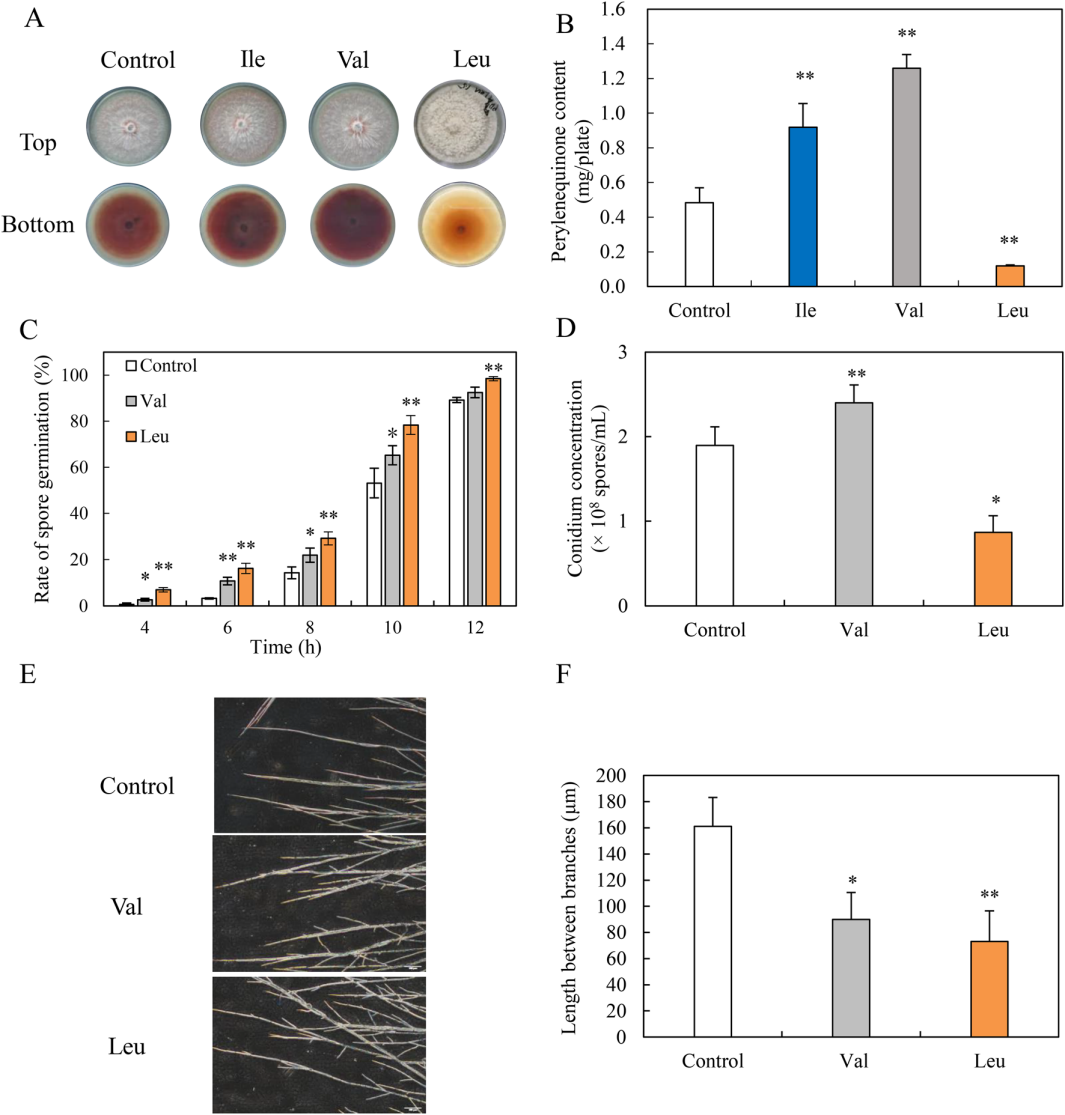
The effects of exogenous BCAAs (l-Ile, l-Val and l-Leu) on growth and perylenequinone production of *S. bambusicola* S8. **A** The effects on red pigments secretion of *S. bambusicola* S8 in PDA plates. **B** The effects on perylenequinone production in plate. The fungus was treated with BCAAs (1.5 g/L) and incubated at 28 °C for 8 days. **C** The spore germination rate. The spores were washed and cultured in a 30% PDB medium containing l-Val or l-Leu (1.5 g/L). **D** Conidium concentration of S8 were measured on day 8. **E** The mycelial morphology was observed (200 ×). **F** Determination of the length of hyphae branching. A small lump (4 × 4 mm) of *S. bambusicola* S8 was transferred from stock slants to petri dishes containing l-Val and l-Leu (1.5 g/L), and then incubated at 28 °C for 3 days. The sterile cover glass was inserted into the edge of the hyphae, and the morphology of the hyphae was observed after 2 days of culture. The culture without l-Val or l-Leu treatment was used as control. Values are mean ± SD from three independent experiments (^*^p < 0.05, ^**^p < 0.01 versus control group)

To further investigate on the effects of BCAAs on fungal perylenequinone production, exogenous l-Val, l-Ile or l-Leu was added respectively at 1.5 g/L to the culture of *S. bambusicola* S8 on PDA medium in 9-cm petri dishes. We observed that the red perylenequinone pigment accumulation was stimulated by the addition of l-Val or l-Ile, but suppressed by l-Leu (Fig. 4A). l-Val and l-Ile promoted the perylenequinone content 2.61- and 1.90-fold over the control respectively, whereas l-Leu treatment decreased the content by 75% (Fig. 4B). l-Val and l-Leu are selected for the subsequent experiment due to their contrasting effects. The conidiation rate increased from 1.89 × 10^8^ to 2.40 × 10^8^ spores/mL under l-Val (1.5 g/L) treatment, while l-Leu treatment inhibited the conidiation to 0.87 × 10^8^ spores/mL (Fig. 4D). However, spore germination was promoted by both treatments (Fig. 4C). Additionally, both treatments reduced the distance between hyphal branches (Fig. 4E, F), while l-Leu treatment resulted in abundant aerial mycelia in plate culture (Fig. 4A). In the mycelium culture, there was no obvious alternation of the fungal biomass by l-Val or l-Leu (Additional file 1: Table S3). However, the individual perylenequinone contents (HA, HC), and EA-EC were all stimulated by l-Val in production medium with Triton X-100, whereas both the intracellular and extracellular perylenequinone production were suppressed sharply by l-Leu (Table 1).

**Table 1:** Effect of l-Val or l-Leu on perylenequinone production in *Shiraia* mycelium cultures^*^

	HA	HC	EA	EB	EC	Total perylenequinone
Intracellular perylenequinones (mg/g DW)						
Control	1.30 ± 0.08	0.59 ± 0.04	0.01 ± 0.001	0.31 ± 0.04	2.07 ± 0.27	4.30 ± 0.36
l-Val	1.83 ± 0.12^*^	0.7 ± 0.17	0.07 ± 0.02^**^	0.37 ± 0.06	2.28 ± 0.06	5.23 ± 0.41^*^
l-Leu	ND	ND	ND	ND	0.48 ± 0.04^**^	0.47 ± 0.04^**^
Extracellular perylenequinones (mg/L)						
Control	50.44 ± 9.10	9.80 ± 1.04	1.92 ± 0.23	12.58 ± 1.92	49.12 ± 6.48	123.96 ± 17.63
l-Val	104.53 ± 11.52^**^	14.63 ± 3.54	3.89 ± 0.67^**^	22.51 ± 4.77^*^	89.99 ± 12.72^**^	233.56 ± 27.57^**^
l-Leu	ND	ND	ND	ND	5.19 ± 0.85^**^	5.19 ± 0.09**

*ND* not detected

^a^l-Val or l-Leu was added at 1.5 g/L on day 3 in the production and maintained at 150 rpm and 28 °C for 8 days. The culture without Val or Leu treatment in the production medium with Triton X-100 was used as control

^*^p < 0.05

^**^p < 0.01 versus control

### Effect of BCAA on acetyl-CoA content and gene expressions involved in central carbon metabolism

**Fig. 5:**
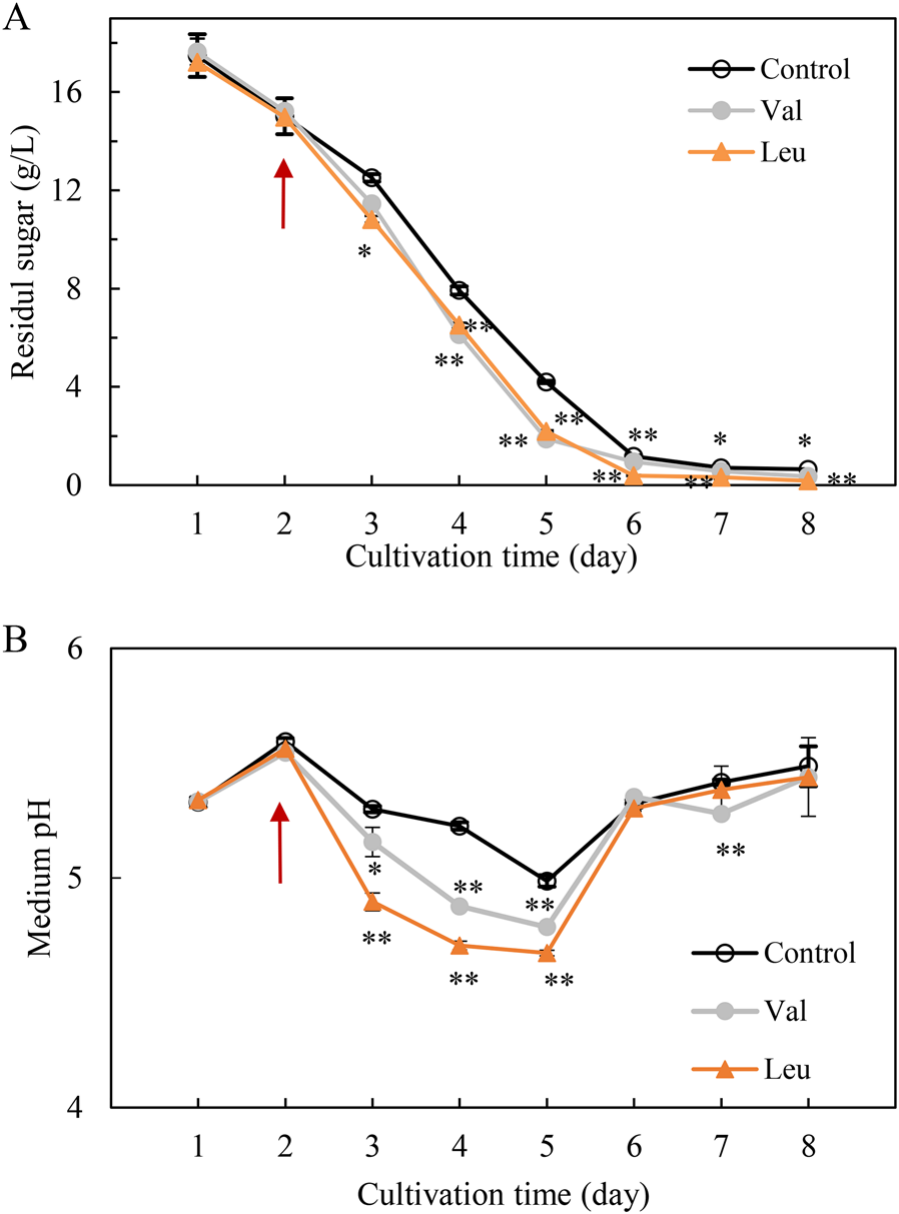
The effects of l-Val and l-Leu on the residual sugar (**A**) and pH value (**B**) in *S. bambusicola* S8 culture. l-Val or l-Leu (1.5 g/L) was added on day 2 of the culture which was maintained at 150 rpm and 28 °C. Arrow indicates the time point of l-Val or l-Leu addition. The culture without l-Val and l-Leu in the production medium with Triton X-100 was used as control. Values are mean ± SD from three independent experiments (^*^p < 0.05, ^**^p < 0.01 versus control group)

**Fig. 6:**
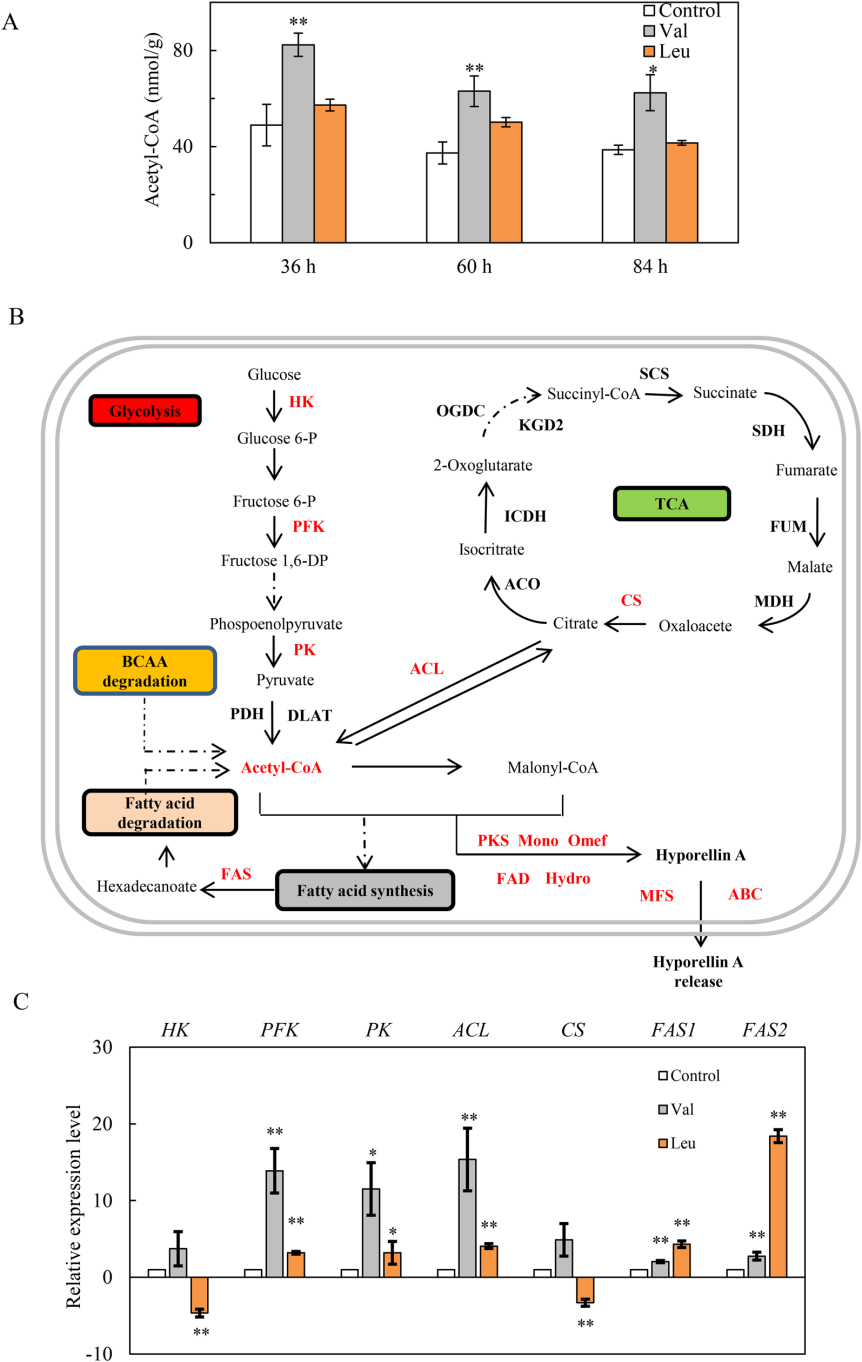
Effect of BCAA on expression of key genes involved in central carbon metabolism and acetyl-CoA content of *S. bambusicola* S8. **A** Acetyl-CoA content in *S. bambusicola* S8. **B** Central metabolic pathway metabolic network diagram containing the glycolysis (EMP), tricaboxylic acid (TCA) cycle, and fatty acid metabolism. Some of steps and compounds are omitted for simplification. **C** The expression of key genes involved in central carbon metabolism. l-Val or l-Leu was added at 1.5 g/L on day 2. *HK* Hexokinase, *PFK* 6-phosphofructokinase, *PK* pyruvate kinase, *ACL* ATP citrate (pro-S)-lyase, *CS* citrate synthase, *FAS1* fatty acid synthase subunit beta and *FAS2* subunit alpha. The culture without l-Val and l-Leu in the production medium with Triton X-100 was used as control. The cultural conditions of *S. bambusicola* S8 was 150 rpm and 28 °C. Values are mean ± SD from three independent experiments (^∗^*p* < 0.05, ^*∗∗*^*p* < 0.01 versus control group)

We found that the residual sugar consumption in the production culture was stimulated after the l-Val or l-Leu treatment (Fig. 5A) and the pH in the medium was significantly decreased (Fig. 5B), indicating a possible regulation on the central carbon metabolism. The acetyl-CoA content after l-Val treatment was raised by 33.3%, 63.6% and 15.6% at 36, 60 and 84 h, respectively (Fig. 6A). However, l-Leu treatment did not cause any significant changes in mycelial acetyl-CoA contents. We investigated on the expression of genes encoding the key enzymes in the glycolysis including hexokinase (HK), 6-phosphofructokinase (PFK) and pyruvate kinase (PK), tricarboxylicacid (TCA) such as ATP citrate (pro-S)-lyase (ACL) and citrate synthase (CS), and fatty acid biosynthesis like fatty acid synthase subunit beta and subunit alpha (FAS1 and FAS2) (Fig. 6B). The qRT-PCR results showed that the expression of most selected genes (*PFK*, *PK*, *ACL*, *FAS1* and *FAS2*) in central carbon metabolism were up-regulated significantly by l-Val or l-Leu (Fig. 6C).

### Effect of BCAAs on expressions of key genes for perylenequinone biosynthesis and HA production

**Fig. 7:**
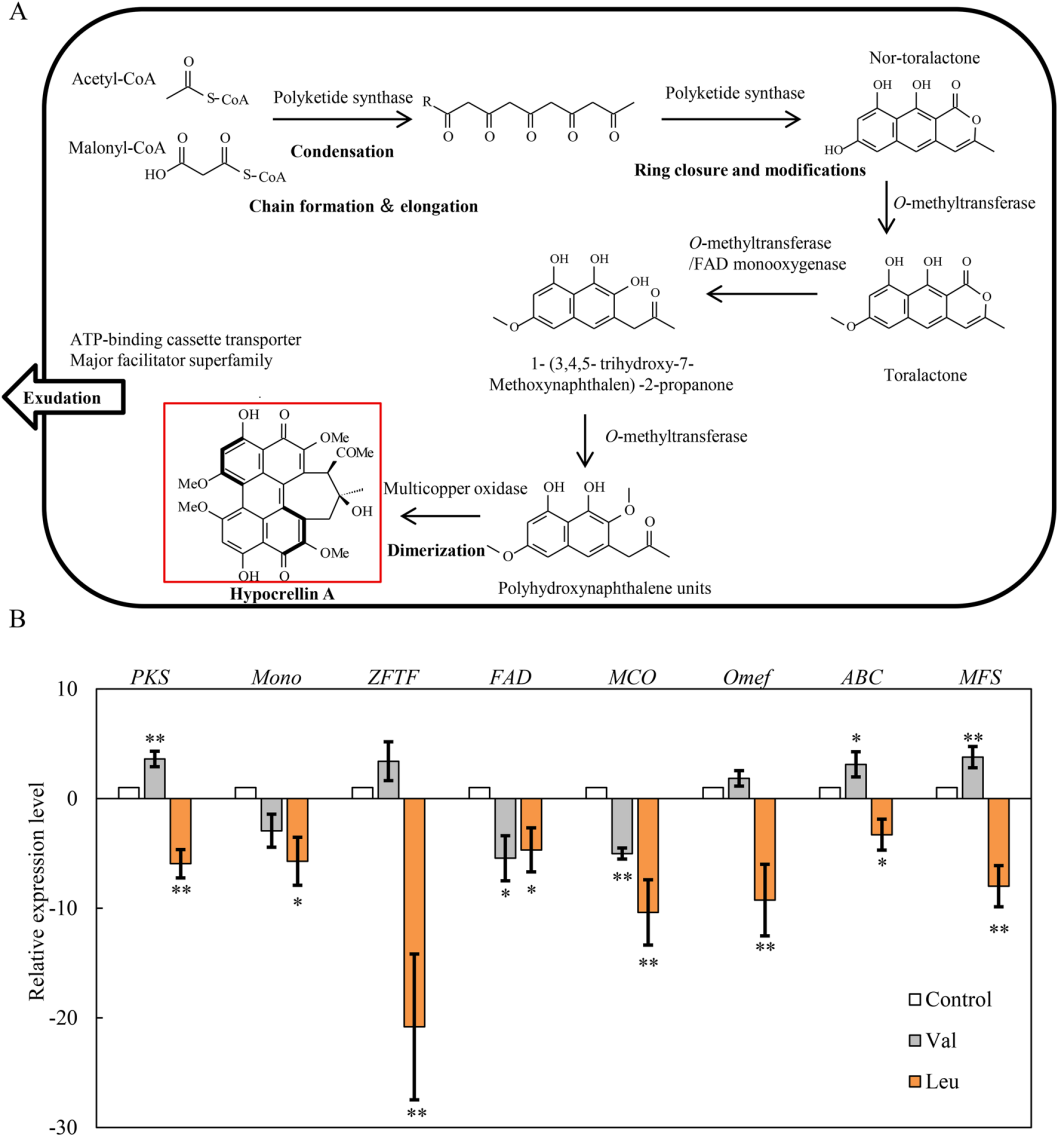
Effect of l-Val or l-Leu on the expression of perylenequinone biosynthetic genes of *S. bambusicola* S8. **A** The proposed perylenequinone biosynthesis pathway. Some of steps and compounds are omitted for simplification. **B** The expression of perylenequinone biosynthetic genes by l-Val or l-Leu added at 1.5 g/L on day 2. The culture without l-Val and l-Leu in the production medium with Triton X-100 was used as control. *PKS* polyketide synthase, *Omef* *O*-methyltransferase, *FAD* FAD/FMN-dependent oxidoreductase, *Mono* monooxygenase, *MCO* multicopper oxidase, *MFS* major facilitator superfamily, *ABC* ATP-binding cassette transporter and *ZFTF* zinc finger transcription factor. Values are mean ± SD from three independent experiments (^∗^*p* < 0.05, ^∗∗^*p* < 0.01 versus control group)

The genome sequencing of *Shiraia* sp. Slf14 was reported by Yang et al. [22] and perylenequinone biosynthetic gene cluster was identified to include polyketide synthase (*PKS*), FAD/FMN-containing dehydrogenase (*FAD*), multicopper oxidase (*MCO*), major facilitator superfamily (*MFS*), ATP-binding cassette  transporter (*ABC*), *O*-methyl-transferase (*Omef*), zinc finger transcription factor (*ZFTF*) and monooxygenase (*Mono*) (Fig. 7A). In the production medium with the l-Val treatment, the expression levels of *PKS*, *ZFTF*, *Omef*, *ABC* and *MFS* were significantly up-regulated by about 3.6-, 3.4-, 1.9-, 3.1- and 3.8-fold respectively of the control group (Fig. 7B). However, the expression levels of all genes were down-regulated by l-Leu treatment (Fig. 7B).

**Fig. 8:**
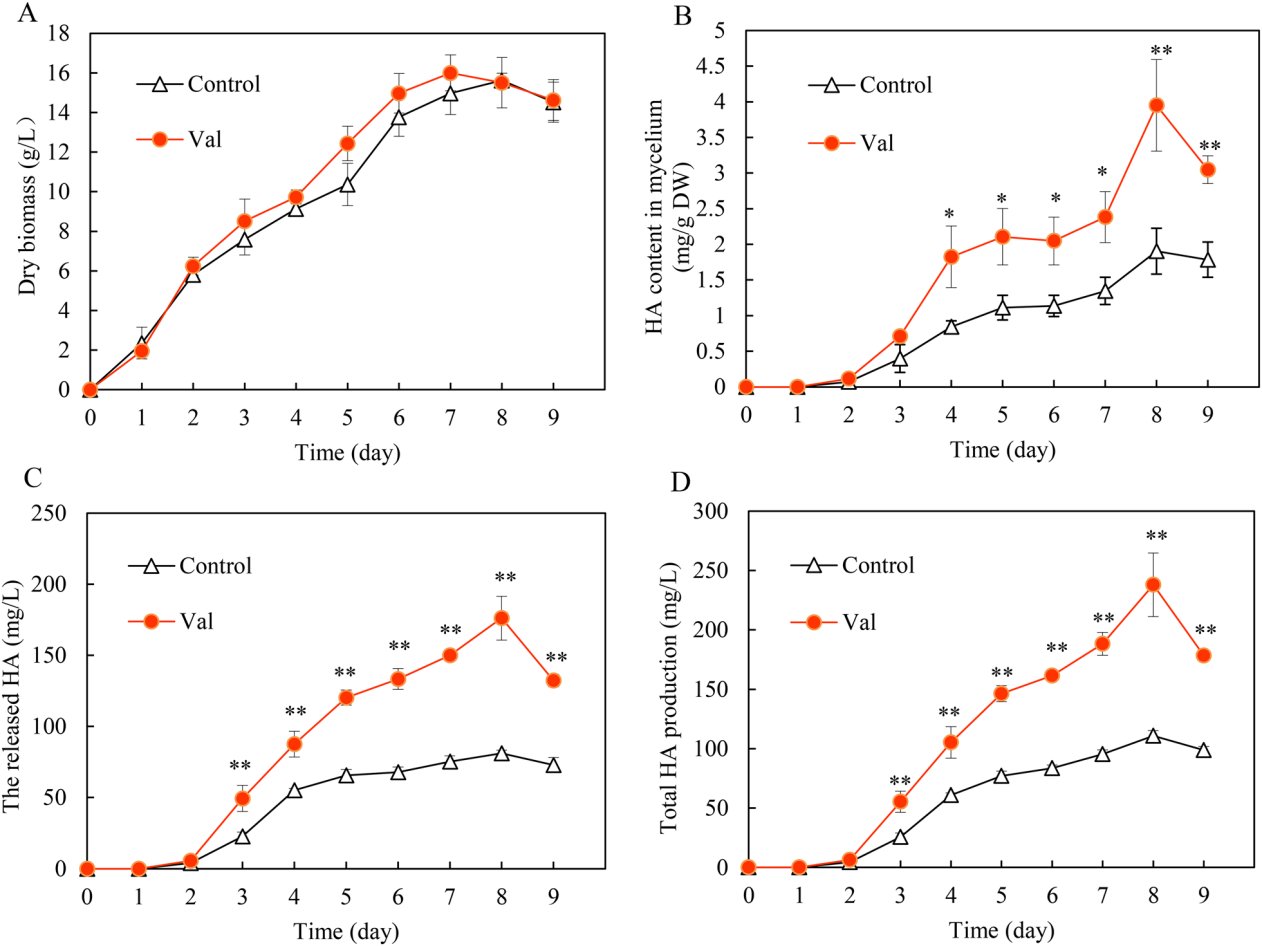
The time-course of fungal biomass (**A**), HA content in mycelium (**B**), the released HA in cultural broth (**C**) and total HA production (**D**) in submerged culture of *S. bambusicola* S8 treated by l-Val. Total HA production refers to the sum of the intracellular and extracellular HA. The fungus was treated with l-Val (1.5 g/L) on day 2. The culture untreated with l-Val in the production medium with Triton X-100 was used as control. Values are mean ± SD from three independent experiments (^*^p < 0.05, ^**^p < 0.01 versus control group)

To optimize l-Val treatment for the enhanced production of HA, a main bioactive perylenequinone in *Shiraia* mycelium culture, the addition time and concentrations were investigated in the production medium with Triton X-100 (Additional file 1: Fig. S1, S2). Although l-Val treatment did not cause alteration on fungal biomass (Additional file 1: Fig. S1A), HA contents in mycelium or in the medium were enhanced after l-Val addition on day 2, 3 (Additional file 1: Fig. S1B, C). The higher production of HA production was achieved on day 2 (Additional file 1: Fig. S1D). When l-Val (1–3 g/L) was added to *Shiraia* mycelium culture on day 2, we also found the fungal biomass did not change (Fig. S2A), but both the intracellular and extracellular HA contents were promoted by l-Val treatment (1–3 g/L) (Additional file 1: Fig. S2 B–D). Simultaneously, the other perylenequinones including HC and EA-EC were stimulated after l-Val treatment at 1.5 g/L on day 2 (Additional file 1: Fig. S3). In the time course study of l-Val treatment, the hypha biomass showed an exponential growth during the cultures and there was no obvious alternation compared to the control group (Fig. 8A). The HA content in mycelium initially maintained a small quantity within 2 days, then increased with time up to day 8 (Fig. 8B). The released HA in cultural broth was also stimulated and reached the peak value on day 8 (Fig. 8C). The highest HA production (237.92 mg/L) reached on day 8, about 2.12-fold over the control (Fig. 8D).

## Discussion

Several genera of plant pathogenic fungi (*Alternaria*, *Cercospora*, *Cladosporium* and *Shiraia*) can produce photoactivated perylenequinone toxins as chemical tools for infection of host plants [23]. perylenequinone toxins such as HA, cercosporin and elsinochrome share a common 3,10-dihydroxy-4,9-perylenequinone chromophore that gives rise to the ability to be activated by visible light to generate ROS including singlet oxide ^1^O_2_, superoxide O_2_^−^, hydrogen peroxide (H_2_O_2_), and the hydroxyl radical (OH^·^) [23]. The pathogenic fungi such as *Shiraia*, *Cercospora* and *Elsinoё* species utilized perylenequinone-induced ROS to cause indiscriminate cellular damages to the host cell membrane within minutes of light exposure, leading to disease development of many economically important plants such as citrus, corn, coffee and soybean as well as vegetable crops [24]. *Shiraia* species are pathogenic fungi to infect more than 10 species of bamboos in China and Japan [25]. The pathogenicity factor for *Shiraia* infection was believed to be ROS damage induced by photoactivated HA [9, 26]. In the present study, both the transcriptomic analysis and exogenous BCAA treatments demonstrated that BCAA could make a regulatory role on *Shiraia* growth and perylenequinone biosynthesis. We can reasonably speculate that BCAA metabolism is closely related to *Shiraia* pathogenicity. BCAAs have been found to play a crucial role in maintaining fungal pathogenicity [27]. For example, dihydroxyacid dehydratase (DHAD) encoded by gene *ilv3* is a key common enzyme in the BCAA biosynthetic pathway of the filamentous fungus *Aspergillus fumigatus*. The *ilv3B* deletion mutant was unable to grow and produce asexual spores in the absence of BCAAs, exhibiting lower virulence in murine infection models [28]. Threonine dehydratase, the first critical enzyme in the Ile biosynthesis in *Fusarium graminearum* played a key role in hyphal growth, conidiation and production of deoxynivalenol, a crucial virulence factor of *F. graminearum* [29]. The further investigation is needed to verify the role of BCAAs on *Shiraia* virulence by using metabolic engineering for BCAAs. This study is the first to assess the physiological role of BCAAs in perylenequinone-producing fungi and provides helpful hints for controlling plant diseases caused by fungal photoactive perylenequinones.

Some fungal photoactive perylenequinones such as hypocrellins have been recently developed as photosensitizers for PDT on cancer and infections clinically [30]. The biotechnological production of perylenequinone in *Shiraia* mycelium culture is becoming a promising alternative to the chemical extraction from the wild fruiting bodies. Recently, various strategies have been applied to promote perylenequinone production, including cultural medium optimization [31, 32], using microbial or abiotic elicitors (La^3+^, surfactant treatment, light and ultrasound stimulation) [16, 17, 33]. Although nitrogen sources including amino acids are required for *Shiraia* growth and development [34], few studies have paid attention to the regulatory effects of amino acids on the fungal secondary metabolites. Recently, Chen et al. [35] reported that L‑arginine at 7 g/L could increase perylenequinone yield of *Shiraia* sp. Slf14 and its natural mutant *Shiraia* sp. Slf14 (w) by 1.51- and 30.52-fold respectively [35]. BCAAs were used to enhance the production of macrocyclic polyketide antibiotic in *Streptomyces* [13]. The production of macrocyclic polyketide metabolite pamamycin, isolated from *S. albus* J1074/R2, was increased by 300% only after exogenous l-Val (3 mM) treatment [36]. In the present study, l-Val treatment (1.5 g/L) enhanced significantly both intracellular and extracellular perylenequinones in *Shiraia* mycelium culture (Fig. 8B, C). The highest HA production (237.92 mg/L) was reached after 6 days of l-Val treatment, about 2.12-fold of the control (Fig. 8D). In our previous study, the enhanced HA production under the stimulation of repeated ultrasound [16], light–dark shift [18], and red light [17], was ranged from 175.53 to 247.67 mg/L. In this study, we reported for the first time that BCAA addition could significantly influence fungal perylenequinone production. Compared with the elicitation techniques reported previously, BCAA treatment have the advantages of higher efficiency for the stimulation and more easily application for large-scale cultures in bioreactors.

The *Shiraia* perylenequinones are biosynthesized via a polyketide pathway [37]. BCAAs degradation was reported to provide precursors for the biosynthesis of macrocyclic polyketide metabolites [38]. Increases in productions of actinorhodin by *S. coelicolor* A3(2) were achieved by overexpressing BCDH for catabolism of BCAAs to provide more acetyl-CoA, a common precursor for all polyketide synthase derived biosynthesis [14]. In this study, more acetyl-CoA was accumulated in *Shiraia* mycelia after the l-Val treatment (Fig. 6A), providing more substrates for perylenequinone biosynthesis. On the other hand, we found that there were more glucose consumption and the decreased pH value in the medium under BCAA treatment (Fig. 5A, B), suggesting possible changes in central carbon metabolism. As we known, the precursors for perylenequinone biosynthesis such as acetyl-CoA and malonyl-CoA are mainly derived from the primary metabolisms including glycolysis (EMP), tricarboxylicacid (TCA) and amino acid metabolism (Fig. 6B). In this study, we found the expression of key genes involved in EMP and TCA was significantly upregulated under the l-Val treatment, which could provide more substrates for perylenequinone biosynthesis (Fig. 6C) [37]. Interestingly, we found the expressions of gene *FAS1* and *FAS2*, the cytosolic metabolic enzymes that catalyze de novo fatty acid (FA) synthesis, were enhanced significantly after the addition of l-Leu, implying the facilitation of FA synthesis and inhibition of perylenequinone biosynthesis (Fig. 6B).

In the perylenequinone biosynthesis, the starter acetyl CoA and malonyl CoA are firstly catalyzed by polyketide synthase of *S. bambusicola* (*SbaPKS*) to a common aromatic polyketide precursor, nor-toralactone, via condensation and decarboxylation (Fig. 7A) [9]. It was reported that expression levels of adjacent genes in perylenequinone biosynthesis gene cluster, such as *Omef* and *MFS* gene, are regulated by the gene *SbaPKS* [9]. In the present study, we found that the expression level of *PKS* was significantly up-regulated by l-Val treatment (Fig. 7B). The expressions of most adjacent genes, including *ZFTF* and *Omef*, were also up-regulated. The major facilitator superfamily (MSF) family transporter and the ATP-binding cassette transporter (ABC) family transporter have been identified to be participated in the hypocrellin transport in *S. bambusicola* [26, 33]. It is noteworthy that l-Val treatment also up-regulated the expression of *ABC* and *MFS*, leading to the released perylenequinones in the medium (Fig. 7B). On the contrary, l-Leu served to suppress or silence the expression of all genes for perylenequinone biosynthesis (Fig. 7B). Taken together with the above results on perylenequinone production, our study suggested that BCAAs could be important factors for fungal perylenequinone biosynthesis.

## Conclusion

In summary, this study presented the first assessment of BCAA roles in perylenequinone-producing fungi. The production of *Shiraia* perylenequinones was stimulated significantly by l-Val, but sharply suppressed by l-Leu addition. The highest HA production (237.92 mg/L) in the mycelium culture was reached after 6 days of l-Val treatment at 1.5 g/L. l-Val addition enhanced the perylenequinone biosynthesis via both promoting the central carbon metabolism for more precursors such as acetyl-CoA and eliciting the expression of perylenequinone biosynthetic genes in later steps for perylenequinone biosynthesis. With the optimization of culture conditions including BCAA addition and the combination with other elicitation strategies, the bioactive perylenequinone production in mycelium cultures could be greatly enhanced. Our findings could provide a basis for understanding the mechanism of BCAA regulation on photoactivated perylenequinone toxins in fungi and provide a new strategy for enhanced biotechnological production of perylenequinone photosensitizer in *Shiraia* mycelium cultures.

## Materials and methods

### Strains and culture conditions

The fungal strain *S. bambusicola* S8 (CGMCC3984) used in this study was isolated in our Lab [16]. The fungus was grown on a potato dextrose agar (PDA) medium at 28 °C for 9 days. The basal medium for seed cultures and mycelium culture contained the following components (per liter): 200 g potato, 20 g glucose, 1 g KH_2_PO_4_, 0.5 g MgSO_4_, 0.5 g KCl, 0.01 g FeSO_4_·7H_2_O, 3 g yeast extract, and 10 g peptone. Triton X-100 at 25 g/L was added to the basal medium as the perylenequinone production medium. The details of the seed culture and mycelium cultures and culture conditions were descripted in our previous report [15]. In submerged mycelium culture, the seed broth (10%, v/v) of *S. bambusicola* S8 was transferred into a 150-mL Erlenmeyer flask containing 50-mL production medium, and incubated in a rotary shaker (ZD-8802, Hualida, Suzhou, China) for 8 days at 28 °C, 150 rpm. BCAAs (Val, Leu and Ile in l-configuration, Yuanye Bio-Technology Co Ltd, Shanghai, China) were used to provide exogenous BCAA treatment. *S. bambusicola* S8 was grown on a potato dextrose agar (PDA) medium containing l-Val, l-Leu and l-Ile at 1.5 g/L respectively at 28 °C for 8 days. The mycelial morphology, conidiation and conidia germination rate were measured. To investigate the effect of l-Val addition time, the mycelium cultures were treated once by l-Val at 1.5 g/L at different time (day 0–6) and HA production was determined on day 8. To determine the optimal dosage for l-Val treatment, lVal (1.0–3.0 g/L) was added on day 2 in the production medium in the 8-day cultures.

### Assessment of differential gene expression and gene annotation

The mycelium samples from mycelium cultures on day 8 in the basal medium (control) and perylenequinone production medium with Triton X-100 were used for transcriptome analysis in our lab [15]. Sequencing data were deposited in the National Center for Biotechnology Information (NCBI) as BioProjectPRJNA323638. The cDNA libraries were sequenced using HiSeqTM2500 platform (Illumina, San Diego, USA). De novo assemble was performed using the Trinity program (version: trinityrnseq_r20131110) [39] and software TGICL [40]. All unigenes were assigned to putative gene description following BLASTX alignment to the databases such as Kyoto Encyclopedia of Genes and Genomes (KEGG, http://www.genome.jp/kegg/) and Gene Ontology (GO, http://www.geneontology.org/) with a cut off *E* value of ≤ 1e^−5^. Gene expression was measured by calculating Fragments Per Kilobase of transcripter Million mapped reads [41]. Thresholds of an adjusted *p*-value ≤ 0.05 and the absolute value of fold change ≥ 2 were selected for DEGs determination [42].

### Determination of intracellular amino acid and acetyl-CoA contents

For amino acid analyses, each sample (0.2 g dry mycelia) was hydrolyzed by an electric heating blast drying oven (GZX-GF-9023-BS, Shanghai, China) with hydrogen chloride (6 mol/L) at 110 °C for 22 h under a nitrogen atmosphere. Then, the solution was filtered through a 0.45 μm membrane filter prior to analysis [43]. The amino acid profiles of each sample were determined by an automatic amino acid analyzer (Hitachi LA8080, Tokyo, Japan).

Acetyl-CoA content was determined using the acetyl-CoA assay kit (Suzhou Comin Biotechnology Co., Ltd., Suzhou, China) with malate dehydrogenase-coupled assay system [44]. Briefly, each sample (0.1 g of fresh mycelia) was ground with liquid nitrogen to obtain a fine powder. Then, the sample was treated with 1 mL of lysis solution and ground. The supernatant (100 μL) was obtained after centrifuging at 8000 g at 4 °C for 10 min and treated with the working liquid (920 μL). The absorbance at 340 nm was measured at 20 s (A1) and 80 s (A2) and the value of ΔA (ΔA = A2 − A1) was calculated. Acetyl-CoA content was expressed as nmol/g fresh weight. Acetyl-CoA (nmol/g) = (1640 × ΔA + 0.012) × 10.

### Measurement of conidiation, hyphal elongation and branching

The conidia were collected in sterile water on day 8 of plate culture and the number of spores was counted using a hemocytometer and a light microscope (CX21, Olympus, Tokyo, Japan). The spores (200 uL, 2 × 10^7^ spores/mL) were washed from 8-day PDA plate and inoculated in a 30% basal medium for germination experiments. The germination rate was determined by randomly counting 500 spores for each sample.

To determine the length between branches, a fungal lump (4 × 4 mm) was transferred from stock slants to petri dishes and then incubated at 28 °C for 3 days. The sterile cover glass was inserted into the edge of the hyphae, and the morphology of the hyphae was observed using an optical microscope (CKX41, Olympus, Japan) at a 200 × magnification after 2 days of culture. To determine the biomass accumulation, the mycelium was harvested on day 8 and filtered through 400-mesh filter membrane (Dongkang, Tianjin, China). The mycelium was washed with sterilized water and dried at 50 °C to constant dry weight (DW).

### Measurement of residual glucose sugar, medium pH in cultural broth

The culture broth (50 mL) was harvested by filtration with 400-mesh filter membrane (Dongkang, Tianjin, China) and then used to determine the content of residual sugar by anthrone test using glucose as the standard [45]. The medium pH in cultural broth was measured by pH electrode meter (FE20, Metteler Toledo, Switzerland).

### Extraction and quantification of perylenequinones

The perylenequinones in PDA plates, mycelia and fermentation broth were extracted following the method described previously [8]. The individual perylenequinones were qualified by a reversephase Agilent 1260 HPLC system (Agilent Co., Wilmington, USA) equipped with the Agilent HC-C18 column (250 × 4.6 mm dimension) with a mobile phase (acetonitrile: water at 65: 35, v/v) at 1 mL/min for 20 min and with UV detection at 465 nm [15]. The sum of HA, HB, HC, EA, EB and EC was taken as the total perylenequinone contents.

### Quantitative real-time PCR

The total RNA of the fungal mycelia was isolated using RNAprep pure Plant Kit (Tiangen, Beijing, China). The 18S ribosomal RNA was used as internal reference gene. Primer sequences used for qRT-PCR are listed in in Additional file 1: Table S4. The method of qRT-PCR was performed according to our previous study [46]. The transcriptional expression levels of genes were calculated from cycle threshold values by using the 2^−△△CT^ method_._

### Statistical analysis

All the experiments were consisted with triplicate independent repeats (ten plates or flasks per replicate). Data were subjected to student’s *t*-test and one-way analysis of variance (ANOVA) with Dunnett’s multiple comparison tests and represented as mean ± standard deviation (SD). The *p* value < 0.05 was considered statistically significant.

## Supplementary Information

**Additional file 1: Table S1.** The biomass and total perylenequinone production of *S. bambusicola* S8 in the basal medium without Triton X-100 and production medium with Triton X-100. **Table S2.** The differentially expressed genes that encoding enzymes involved in BCAA biosynthesis and degradation. **Table S3.** Effects of l-Val and l-Leu on biomass of *S. bambusicola* S8. **Table S4.** Primers and relevant information of reference and target genes. F: forward primer, R: reverse primer. **Figure S1.** Effects of introducing time of l-Val on fungal biomass (A), HA content in mycelium (B), the released HA in cultural broth (C) and total HA production (D) in submerged culture of *S. bambusicola* S8. Total HA production refers to the sum of the intracellular and extracellular HA. The culture was treated with l-Val at 1.5 g/L on different time points and incubated at 150 rpm and 28 °C for 8 days. The culture untreated with l-Val in the production medium with Triton X-100 was used as control. Values are mean ± SD from three independent experiments (^*^p < 0.05, ^**^p < 0.01 versus control group). **Figure S2.** Effects of l-Val treatment at different concentrations on fungal biomass (A), HA content in mycelium (B), the released HA in cultural broth (C) and total HA production (D) in submerged culture of *S. bambusicola* S8. Total HA production refers to the sum of the intracellular and extracellular HA. The fungus was treated with l-Val at different concentrations on day 2 and incubated at 150 rpm and 28 °C for 8 days. The culture untreated with l-Val in the production medium with Triton X-100 was used as control. Values are mean ± SD from three independent experiments (^*^p < 0.05, ^**^p < 0.01 versus control group). **Figure S3.** The HPLC chromatogram of perylenequinone production in *Shiraia* mycelium culture under the l-Val treatment at 1.5 g/L on day2. The culture untreated with l-Val in the production medium with Triton X-100 was used as control.

## Data Availability

All data generated or analyzed during this study are included in this published article and its Additional files.
