# Supplementary material for: Effects of branched-chain amino acids on Shiraia perylenequinone production in mycelium cultures
Source: Microb Cell Fact. 2023 Mar 24;22:57. doi: 10.1186/s12934-023-02066-6 (PMC10039612; doi:10.1186/s12934-023-02066-6)
Supplement: Supplementary file 1 — Additional file 1: Table S1. The biomass and total perylenequinone production of S. bambusicola S8 in the basal medium without Triton X-100 and production medium with Triton X-100. Table S2. The differentially expressed genes that encoding enzymes involved in BCAA biosynthesis and degradation. Table S3. Effects of l-Val and l-Leu on biomass of S. bambusicola S8. Table S4. Primers and relevant information of reference and target genes. F: forward primer, R: reverse primer. Figure S1. Effects of introducing time of l-Val on fungal biomass (A), HA content in mycelium (B), the released HA in cultural broth (C) and total HA production (D) in submerged culture of S. bambusicola S8. Total HA production refers to the sum of the intracellular and extracellular HA. The culture was treated with l-Val at 1.5 g/L on different time points and incubated at 150 rpm and 28 °C for 8 days. The culture untreated with l-Val in the production medium with Triton X-100 was used as control. Values are mean ± SD from three independent experiments (*p < 0.05, **p < 0.01 versus control group). Figure S2. Effects of l-Val treatment at different concentrations on fungal biomass (A), HA content in mycelium (B), the released HA in cultural broth (C) and total HA production (D) in submerged culture of S. bambusicola S8. Total HA production refers to the sum of the intracellular and extracellular HA. The fungus was treated with l-Val at different concentrations on day 2 and incubated at 150 rpm and 28 °C for 8 days. The culture untreated with l-Val in the production medium with Triton X-100 was used as control. Values are mean ± SD from three independent experiments (*p < 0.05, **p < 0.01 versus control group). Figure S3. The HPLC chromatogram of perylenequinone production in Shiraia mycelium culture under the l-Val treatment at 1.5 g/L on day2. The culture untreated with l-Val in the production medium with Triton X-100 was used as control. [file 12934_2023_2066_MOESM1_ESM.pdf]

Additional file 1

Effects of branched-chain amino acids on *Shiraia* perylenequinone production in mycelium cultures

Wen Hao Shen, Rui Peng Cong, Xin Ping Li, Qun Yan Huang, Li Ping Zheng and Jian Wen Wang

✉ Jian Wen Wang

jwwang@suda.edu.cn; bcjwwang@gmail.com

Table S1 The biomass and total PQ production of *S. bambusicola* S8 in the basal medium and production medium*

	Dry biomass (g/L）	Total PQ production (mg/L)
Basal medium	16.21±1.17	58.96±5.03
Production medium	17.29±0.40	420.22±49.27**

* *S. bambusicola* S8 was cultured in the basal medium and production medium and maintained at 150 rpm and 28℃ for 8 days. Values are mean±SD from three independent experiments (**p < 0.01).

Table S2 The differentially expressed genes that encoding enzymes involved in BCAA biosynthesis and degradation.

Unigene ID	Fold change[a]	*p* value[b]	Description
**BCAA biosynthesis**			
comp14503_c0_seq1	1.71	0.02809268	Acetolactate synthase (ALS)
comp10799_c0_seq4	2.73	0.03177667	Branched chain amino acid aminotransferase(BCAT)
**BCAA degradation**			
comp10799_c0_seq4	2.73	0.03177667	Branched chain amino acid aminotransferase(BCAT)
comp3266_c0_seq1	45.93	$6.66E^{-11}$	Enoyl-CoA hydratase(ECHS)
comp16002_c0_seq1	6.40	$1.21E^{-5}$	Aldehyde dehydrogenase(ALDH)
comp6091_c0_seq1	2.69	0.029398166	3-hydroxyisobutyrate dehydrogenase(HIBADH)
comp2536_c0_seq2	2.31	0.027267344	Aldehyde dehydrogenase(ALDH)
comp13980_c0_seq1	2.01	0.002002058	Acyl-CoA dehydrogenase(ACAD)
comp6291_c0_seq2	1.95	0.010035825	Aldehyde dehydrogenase(ALDH)
comp16558_c0_seq1	-50.00	0.0098073	Betaine aldehyde dehydrogenase(ALDH)
comp2561_c0_seq1	-33.33	$1.59E^{-14}$	Betaine-aldehyde dehydrogenase(ALDH)
comp15328_c0_seq1	-14.29	0.006054954	Branched chain keto acid dehydrogenase E1 beta polypeptide(BCKDHA)
comp15804_c0_seq1	-11.11	$2.26E^{-5}$	Methylglutaconyl-CoA hydratase(AUH)
comp14759_c0_seq1	-11.11	0.001202752	Branched chain alpha-keto acid dehydrogenase complex, alpha subunit(BCKDHB)
comp11425_c0_seq1	-10.00	0.006404551	3-ketoacid-CoA transferase(OXCT)
comp4910_c0_seq2	-10.00	$1.02E^{-4}$	Dihydrolipoamide branched chain transacylase(DBT)
comp15368_c0_seq1	-9.09	$4.61E^{-6}$	Methylcrotonoyl-CoA carboxylase subunit beta (MCC2)
comp15830_c0_seq1	-8.33	$1.02E^{-5}$	Isovaleryl-CoA dehydrogenase(IVD)
comp13289_c0_seq6	-5.56	0.019236363	Acetoacetyl-CoA synthetase(AACS)
comp6885_c0_seq2	-5.00	$9.76E^{-5}$	Enoyl-CoA hydratase(ECHS1)
comp16074_c0_seq1	-4.76	0.001640936	3-methylcrotonyl-CoA carboxylase subunit alpha(MCC1)
comp15188_c0_seq1	-3.57	0.001573074	Acetyl-CoA acetyltransferase(ACAT1)
comp15605_c0_seq1	-2.70	0.005197635	Hydroxymethylglutaryl-CoA lyase(Hmgl)
comp15451_c0_seq1	-2.63	0.014214445	3-hydroxyisobutyryl-CoA hydrolase(HIBCH)
comp14566_c0_seq1	-2.38	0.0050095	Acetyl-Coenzyme A acyltransferase 1(ACAA1)
comp5864_c0_seq1	-2.13	0.015305063	Acetyl-CoA acetyltransferase(ACAT2)
comp2389_c0_seq2	-1.89	0.024897424	Betaine-aldehyde dehydrogenase(ALDH)

[a] Foldchange, ratio (S1/S2). S1, the FPKM value of a unigene in production medium with Triton X-100; S2, the FPKM value of a unigene in the basal medium without Triton X-100.
[b] *p*-value≤ 0.05 is mean statistically significant.
The cultural conditions of *S. bambusicola* S8 was 150 rpm and 28°C and the samples were collected from the basal medium and production medium on day 8.

Table S3 Effects of L-Val and L-Leu on biomass of *S. bambusicola* S8*

	Biomass (g/L）
Control	12.90±0.79
L-Val	13.69±0.42
L-Leu	13.52±0.83

* 1.5 g/L L-Val or L-Leu was added on day 3 in the production medium and maintained at 150 rpm and 28℃ for 8 days. The culture without L-Val or L-Leu treatment was used as control ($^{*}p < 0.05$ and, $^{**}p < 0.01$ versus control).

Table S4 Primers and relevant information of reference and target genes. F: forward primer, R: reverse primer.

Genes symbol	Gene name	Sequence
18S	Reference gene	F: GAAAGTTAGGGGATCGAAGA<br>R: TAGTCGGCATAGTTTACGGT
**PQ bioysnthesis**		
PKS	Polyketide synthase	F: TGCTGAGGTAGCAGTCAAGC<br>R: TTATGCTACGGTCGTCGCTC
FAD	FAD/FMN-containing dehydrogenase	F: TGTGACCGCCATCACCTTAC<br>R: TTGTCGTATGGGTGGGAAGC
MCO	Multicopper oxidase	F: TATGGCGCTACGAGTGGAC<br>R: ACTCCCTGGCCGATAACGTA
Omef	O-methyltransferase	F: GAACTACCTGAAGGCACGCT<br>R: GCTCGGAAGGATACTCGCTC
ZFTF	Zinc finger transcription factor	F: GAACACCGTCGCAAGATTCG<br>R: TCATTGGCATCGCTTGGAGT
Mono	Salicylate 1-monooxygenase	F: TCTCGGGGAATTATGGCACG<br>R: ACAACCGTTCTCGCATCAGT
ABC	ATP-binding cassette	F: GACTTGAGCCTATCCGCCTC<br>R: AGAGTCGCCTCTGTGATCCT
MFS	Major facilitator superfamily	F: TCCCGTAGCCTTGCTTTCTG<br>R: CCGGCTTCTTCTTGACGCTA
**Glycolytic pathway (EMP)**		
comp6515_c0_seq1	Hexokinase (HK)	F: GGACCAATTTGCCCCCAAAC<br>R: TTTTCCGCCATCCAGTCGAA
comp2690_c0_seq1	6-phosphofructokinase (PFK)	F:CCTGAACGCACTCATCCAGT<br>R:CAAACCAGCAACAGTCGCAA
comp14878_c0_seq1	Pyruvate kinase (PK)	F: CCTCCCAGCCAGGTTTTCAT<br>R:GACAACATGACGCAGTCAGC
**Tricarboxylicacid (TCA)**		
comp11077_c0_seq3	ATP citrate (pro-S)-lyase (ACL)	F: TGCTGAGGAGTTCAGCAAGG<br>R:GACACGAAGATCGGGGTTGT
comp15045_c0_seq1	Citrate synthase (CS)	F: TCTGTACCTTGCTCTGCACG<br>R:ACTTCTTGAGCAGCCAGACC
**Fatty acid biosynthesis**		
comp2921_c0_seq2	Fatty acid synthase subunit beta（FAS1)	F: AAGCGGTTGACAAAGCGAAC<br>R:TCAGTGCTGCAAGATTCGGT
comp15071_c0_seq1	Fatty acid synthase subunit alpha （FAS2)	F: ATGTCTCGTCCCACAACCAC<br>R: GACTCCTGCCACTGCTTGAT

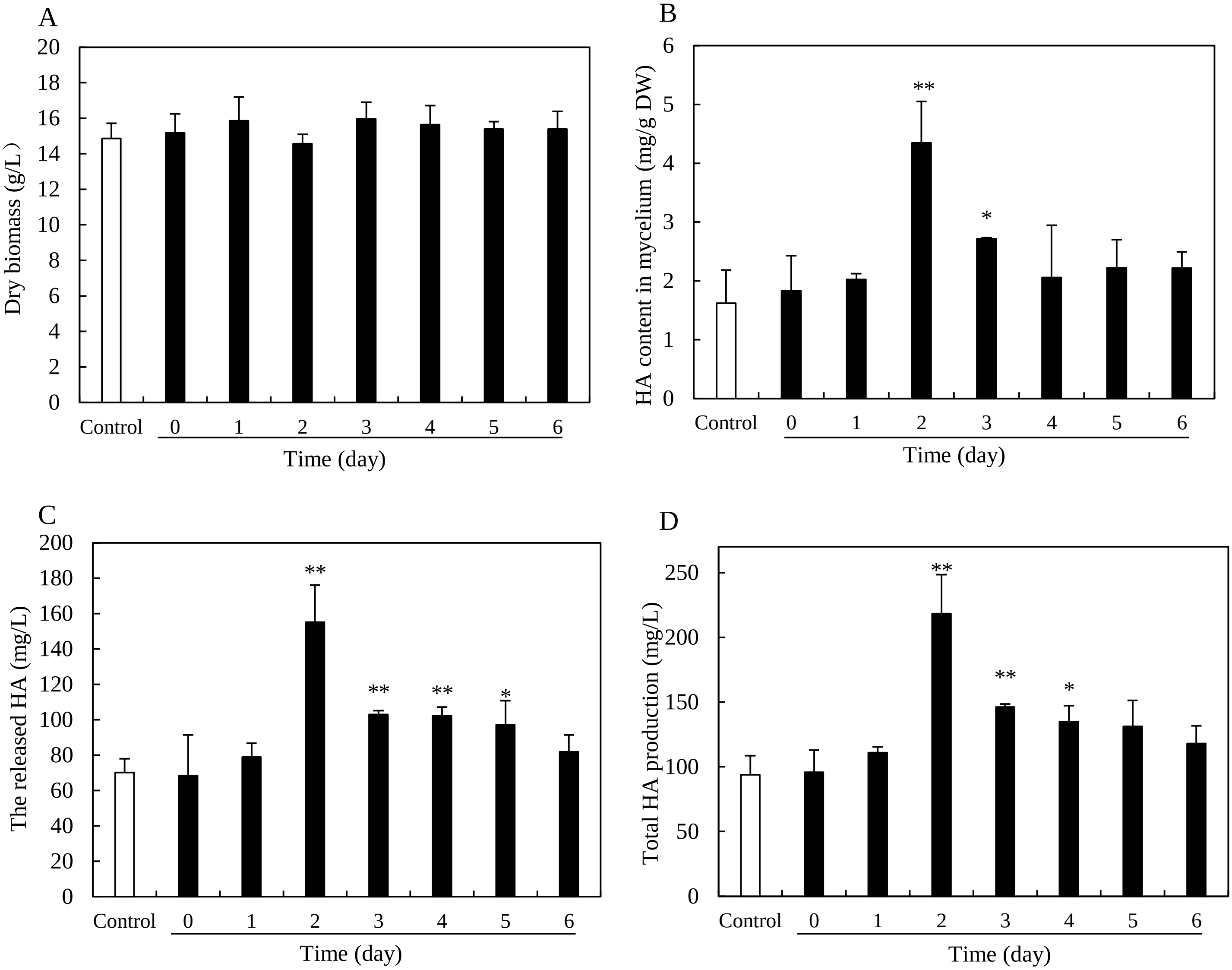

Fig. S1 Effects of introducing time of L-Val on fungal biomass (A), HA content in mycelium (B), the released HA in cultural broth (C) and total HA production (D) in submerged culture of *S. bambusicola* S8. Total HA production refers to the sum of the intracellular and extracellular HA. *S. bambusicola* S8 was treated with L-Val at 1.5 g/L on different time points and incubated at 150 rpm and 28℃ for 8 days. The culture untreated with L-Val in the production medium was used as control. Values are mean ± SD from three independent experiments. ($^{*}p < 0.05$, $^{**}p < 0.01$ versus control group).

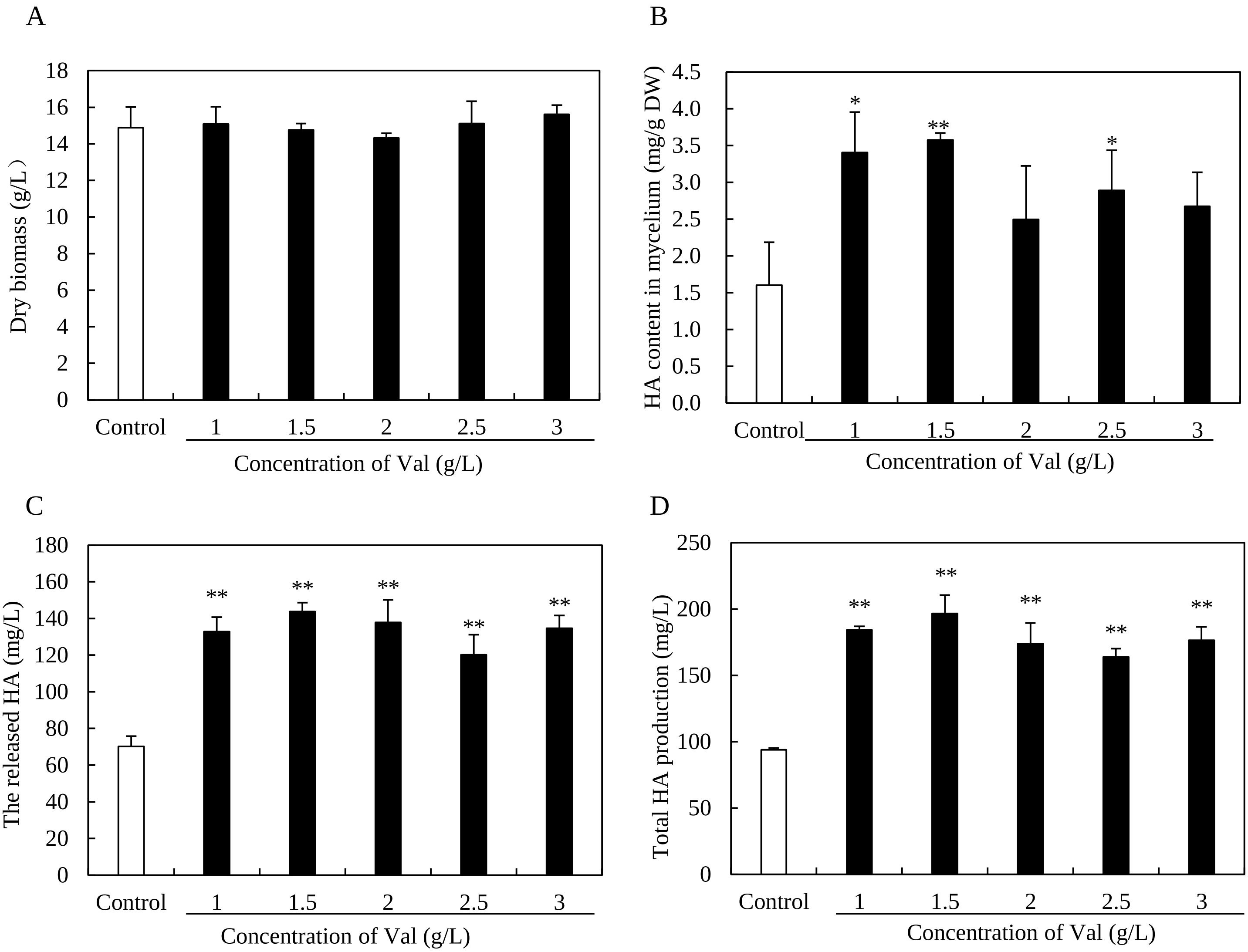

Fig. S2 Effects of L-Val treatment at different concentrations on fungal biomass (A), HA content in mycelium (B), the released HA in cultural broth (C) and total HA production (D) in submerged culture of *S. bambusicola* S8. Total HA production refers to the sum of the intracellular and extracellular HA. *S. bambusicola* S8 was treated with L-Val at different concentrations on day 2 and incubated at 150 rpm and 28℃ for 8 days. The culture untreated with L-Val in the production medium was used as control. Values are mean ± SD from three independent experiments. ($^{*}p < 0.05$, $^{**}p < 0.01$ versus control group).

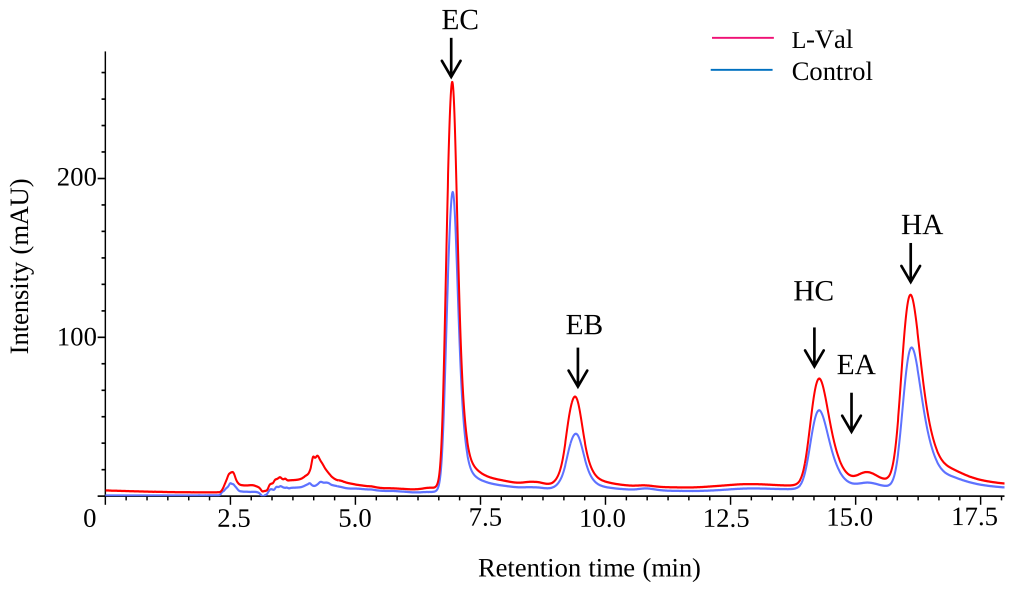

Fig. S3 The HPLC chromatogram of perylenequinone production in *Shiraia* mycelium culture under the L-Val treatment at 1.5 g/L on day 2. The culture without L-Val in the production medium was used as control.
